# 1232. Incorporation of Theory to Develop and Implement a Multi-Faceted Antimicrobial Stewardship Intervention for Hospitalized Adults with Bacteriuria

**DOI:** 10.1093/ofid/ofad500.1072

**Published:** 2023-11-27

**Authors:** Emily Black, Dianne M MacLean, Madison Bell, Paul Bonnar, Andrea J Kent, Kim A Abbass, Valerie Murphy, Heather L Neville, Tasha Ramsey, Melissa Helwig, Olga Kits, Samuel G Campbell, Keri Coulson, Constance LeBlanc, B Lynn Johnston, Ingrid Sketris

**Affiliations:** Dalhousie University, Halifax, Nova Scotia, Canada; Nova Scotia Health Region, Halifax, Nova Scotia, Canada; Dalhousie University, Halifax, Nova Scotia, Canada; Dalhousie University, Halifax, Nova Scotia, Canada; Nova Scotia Health, Halifax, Nova Scotia, Canada; Nova Scotia Health, Halifax, Nova Scotia, Canada; Nova Scotia Health, Halifax, Nova Scotia, Canada; Nova Scotia Health, Halifax, Nova Scotia, Canada; Nova Scotia Health, Halifax, Nova Scotia, Canada; Dalhousie University, Halifax, Nova Scotia, Canada; Nova Scotia Health, Halifax, Nova Scotia, Canada; Dalhousie University, Halifax, Nova Scotia, Canada; CEHHA/NSHEALTH, TRUR0, Nova Scotia, Canada; Dalhousie University, Halifax, Nova Scotia, Canada; Dalhousie University/NSHA, Halifax, Nova Scotia, Canada; Dalhousie University, Halifax, Nova Scotia, Canada

## Abstract

**Background:**

Inappropriate treatment of bacteriuria is commonly reported. While evidence to support antimicrobial stewardship (AMS) interventions has been published, few studies justify intervention components or incorporate theory into designing interventions. The objective of the study was to develop a theory-informed multifaceted AMS intervention to improve management of bacteriuria in adults admitted to hospital.

**Methods:**

We used the 4-step approach described by French and colleagues to develop a theory informed intervention. A systematic review of AMS interventions to improve antibiotic use for bacteriuria was completed. In addition, local barriers to improving antimicrobial use in hospitalized adults with bacteriuria were assessed through a qualitative study using focus groups with health care providers. Barriers identified through the qualitative study were mapped to the Theoretical Domains Framework and the COM-B model then linked to the Behaviour Change Wheel. Published literature, focus group results, and practical considerations were used by our team to identify and rank possible solutions. Consensus on which interventions to implement locally was achieved using the Nominal Group Technique.

**Results:**

Ten interventions that could address local challenges with antimicrobial prescribing for bacteriuria were identified. The highest-ranking interventions were audit and feedback (to individuals or teams), active educational sessions, development of clinical order sets, and incorporating clinical decision support with culture results. A multifaceted intervention that included monthly audit and feedback on management of bacteriuria to multidisciplinary teams in combination with case-based virtual education sessions was developed and is currently being piloted at four tertiary and community hospitals.

**Conclusion:**

Use of theory to identify local barriers and facilitators to improving antimicrobial use in combination with evidence and practical considerations should be incorporated into design and implementation of AMS interventions. Further work will evaluate impact of this theory-informed AMS intervention on antimicrobial prescribing for bacteriuria in hospitalized adults.

**Disclosures:**

**Emily Black, BSc(Pharm), PharmD**, Drug Evaluation Alliance of Nova Scotia: Grant/Research Support|Research Nova Scotia: Grant/Research Support **Paul Bonnar, MD**, BioMerieux: Honoraria|Paladin Labs: Honoraria **Samuel G. Campbell, FRCP (Edin)**, Astra Zeneca: Board Member

